# Investigating the safety and compliance of using csDMARDs in rheumatoid arthritis treatment through face-to-face interviews: a cross-sectional study in China

**DOI:** 10.1007/s10067-020-05458-w

**Published:** 2020-10-15

**Authors:** Jiaying Sun, Siming Dai, Ling Zhang, Yajing Feng, Xin Yu, Zhiyi Zhang

**Affiliations:** 1 Department of Rheumatology and Immunology, First Affiliated Hospital of Harbin Medical University, Harbin, 150086 Heilongjiang China; 2 Shanghai Roche Pharmaceuticals Ltd., Shanghai, 201203 China

**Keywords:** Adverse events, Compliance, csDMARD, Methotrexate, Questionnaire, Rheumatoid arthritis

## Abstract

Rheumatoid arthritis (RA) significantly impacts the health of Chinese patients. Conventional synthetic disease-modifying antirheumatic drugs (csDMARDs) are used as the standard treatment for patients with RA. However, Chinese patients with RA have reported poor compliance with csDMARDs. This study aims to better understand the safety and compliance of using csDMARDs in RA treatment. Face-to-face interviews were conducted by questionnaires on safety and compliance of csDMARDs in 400 patients with RA and 100 rheumatologists from 13 cities in China. Rheumatologists were from Tier 3 Class A hospitals with independent rheumatology departments, who admitted more than 30 patients with RA per week. All patients were diagnosed for > 3 months before the survey and had been treated with csDMARDs for > 3 months. The incidence of adverse events (AEs) that attributed to csDMARDs estimated by rheumatologists was lower than that reported by patients for all four prescribed csDMARDs. Also, types of common AEs in rheumatologist’s perception differed from those in the patient’s report. Only 86% (116/135) of patients claimed they notified their rheumatologist about AEs, and 40.8% (150/368) of patients did not strictly adhere to their prescribed treatment. Reasons why patients were not compliant with their treatment, other than AEs, included symptoms being less severe, travel, and busy working life/business trips. This study revealed gaps in perceptions of csDMARDs-related AEs and medication adherence between rheumatologists and patients. These findings suggested adequate doctor-patient communications, and considerations of multiple real-world situations may improve adherence in the treatment of RA patients.

**Table:** 

**Key Points** *• This study identified gaps in rheumatologists’ perception of the prevalence and type of AEs experienced by their patients, which could potentially help them improve their patients’ compliance with treatment.*

**Electronic supplementary material:**

The online version of this article (10.1007/s10067-020-05458-w) contains supplementary material, which is available to authorized users.

## Introduction

Rheumatoid arthritis (RA) is a chronic autoimmune condition associated with gradual damage and disability of joints [1]. RA affects 0.24% of the global population and 0.3–1% of the population in developed countries [2, 3]. In China, the prevalence of RA is 0.2–0.37%, with a female to male patient ratio of 6:1 [4, 5].

The standard clinical treatment for patients with RA is the administration of disease-modifying antirheumatic drugs (DMARDs) [6]. These in turn can be classified into conventional synthetic DMARDs (csDMARDs), biological DMARDs (bDMARDs), and targeted synthetic DMARDs (tsDMARDs) [7]. csDMARDs include methotrexate (MTX), leflunomide (LEF), and sulfasalazine (SASP; also known as salazosulfapyridine); bDMARDs consist of inhibitors targeting T cells (abatacept), B cells (rituximab), interleukin-6 (IL-6; tocilizumab), and tumor necrosis factor (e.g., adalimumab and etanercept); and tsDMARDs include the Janus kinase inhibitors, tofacitinib, and baricitinib [7].

MTX is recommended by the 2019 European League Against Rheumatism (EULAR) guidelines as first-line treatment for RA, as monotherapy, and in combination with other drugs [7]. MTX is the anchor drug for RA treatment because of its efficacy, safety profile, flexible administration (the mode of administration and dose can be individualized), and low cost (compared with other DMARDs), as well as the extensive clinical experience and familiarity with this drug among rheumatologists [7, 8]. Studies have shown that MTX reduces comorbidities and mortality in RA [9, 10]. LEF or SASP should be considered for first-line treatment in cases where patients are contraindicated or have early intolerance to MTX [7]. In addition, EULAR guidelines recommend bDMARDs and tsDMARDs to be used in combination with a csDMARD. IL-6 pathway inhibitors and tsDMARDs may have some advantages (e.g., efficacy and alternative modes of administration) over other bDMARDs in patients who are unable to use csDMARDs as co-medication [7, 11, 12].

The treat-to-target recommendations for RA not only aim for remission or low disease activity but also for optimizing the patient’s quality of life [13]. The 2019 EULAR guidelines recommend monitoring active RA every 1–3 months using a composite assessment of disease activity that comprises joint counts and American College of Rheumatology (ACR)-EULAR definitions for the remission [7, 8, 14]. EULAR also advocates for the timely adjustment of therapy if there is no improvement in a patient’s condition at 3 months or if the target has not been reached at 6 months.

Nonetheless, the full therapeutic effect of medications can only be actualized if patients reasonably comply with the prescribed treatment [15]. It is believed that 33–50% of all drugs prescribed for chronic conditions are not taken as recommended by physicians [16]. Possible reasons for poor compliance with treatment include side effects related to the treatment and insufficient communication between patients and physicians. A meta-analysis of studies on patients with chronic conditions reported that many patients had significant doubts, unanswered questions, and apprehension about their treatment plans, indicating a patient-physician disconnect in terms of their respective views on the need for treatment [17]. Compliance rates (defined as > 80% compliance with the prescribed treatment) for patients with RA in China ranged from 38.6 to 80.2% in two studies [18, 19]. In another Chinese study, patient-reported csDMARDs adherence (also defined as > 80% compliance with the prescribed treatment) was 38% [20]. Common side effects of csDMARDs are nausea, diarrhea, alopecia, and rash [21]. The side effects of MTX, which also include post-treatment fatigue, headaches, and rheumatoid nodule formation, can be reduced with folic acid supplementation [22]. It is believed that if common side effects are not monitored, patients may either stop medication without consulting their physicians or start taking alternative medicines [23]. However, there is a limited understanding of rheumatologists’ perceptions of adverse events (AEs) from csDMARD use in China.

In this study, we conducted interviews across China with rheumatologists, as well as patients with RA. We first investigated their respective perceptions of AEs arising from csDMARD use, and then explored the reasons for poor patient compliance to csDMARDs. Results from this study will be helpful for rheumatologists to improve their patients’ compliance with RA treatment.

## Materials and methods

Between 14 November and 11 December 2018, face-to-face interviews were conducted with 400 patients with RA, as well as 100 rheumatologists from 13 cities in China: Beijing, Shanghai, Guangzhou, Hangzhou, Chengdu, Fuzhou, Shenyang, Wuhan, Xi’an, Nanjing, Jinan, Zhengzhou, and Changsha, which covered both first-tier and second-tier cities. Rheumatologists were from Tier 3 Class A hospitals with independent rheumatology departments, and there were at most two rheumatologists from the same hospital. The inclusion criteria for rheumatologists were as follows: had ≥ 5 years’ experience as attending physicians, deputy chief physicians or chief physicians, have attended to > 30 patients with RA per week, and routinely made decisions about the course of treatment for patients with RA. All patients were aged ≥ 18 years, diagnosed with RA for > 3 months prior to the survey, and had been treated with csDMARDs for > 3 months. Patients who had stopped csDMARD therapy for < 3 months were still eligible to participate in the survey. The ratio of physicians and patients was controlled at 1:4 in the same hospital. Informed consent was obtained from all study participants at the enrollment.

The face-to-face interviews with both patients and rheumatologists were facilitated via patient- or rheumatologist-specific questionnaires. In the patient questionnaire (Online Resource 1), each patient was asked whether he/she (a) had experienced any AEs while receiving treatment for RA and to select the AE(s) from a list, and (b) had taken csDMARDs in strict accordance with the doctor’s advice and to indicate any reasons for non-compliance. The questionnaire for rheumatologists (Online Resource 2) asked each physician to estimate, based on individuals clinical experience, the following parameters for patients with RA who received csDMARDs: (a) incidence of AEs, (b) circumstances under which rheumatologists considered AEs as serious, (c) the proportion of patients who would not comply with treatment plans, and (d) the ideal versus actual average dosage of MTX and LEF prescribed to Chinese patients with RA.

Patient-reported and rheumatologist-estimated data were analyzed using SAS Studio 3.7 (SAS Institute, Inc., Cary, NC, USA). All analyses were descriptive.

## Results

### Participant characteristics

The ratio of female to male patients who participated in this study was 2:1. MTX was the most commonly prescribed csDMARD, followed by LEF (50.5% and 43.0%, respectively). The most prevalent comorbidities were hypertension (42.3%), diabetes (18.3%), and hyperlipidemia (14.3%).

Per the estimate of rheumatologists, a median of 40 outpatients was seen per week with 20% (median) of patients were newly diagnosed with RA. The most prescribed regimen for newly diagnosed patients with RA was one csDMARD plus nonsteroidal anti-inflammatory drugs (25.0%), followed by a combination of two csDMARDs (22.5%). The characteristics of rheumatologists and patients in the study are shown in Table 1.

**Table 1:** Participant characteristics

	Participants
Patient characteristics	*N* = 400
Sex (female), *n* (%)	269 (67.3)
Age (years), mean (SD)	56.1 (10.9)
BMI (kg/m^2^), mean (SD)	23.2 (3.2)
Prescribed csDMARDs, *n* (%)^a^	
MTX	202 (50.5)
LEF	172 (43.0)
SASP	35 (8.8)
HCQ	55 (13.8)
Comorbidities present, *n* (%)	
Hypertension	169 (42.3)
Diabetes	73 (18.3)
Hyperlipidemia	57 (14.3)
Coronary heart disease	43 (10.8)
Respiratory diseases	37 (9.3)
Chronic renal disease	14 (3.5)
Chronic liver disease	10 (2.5)
Stroke	7 (1.8)
Osteoporosis	4 (1.0)
Tumor(s)	4 (1.0)
Anemia	1 (0.3)
Other rheumatic diseases	23 (5.8)
Daily drug treatment	
Types of drugs, median (IQR)	3.0 (3.0)
Number of tablets taken, median (IQR)^b^	6.0 (6.0)
Rheumatologist characteristics	*N* = 100
Rheumatologist seniority, *n* (%)	
Attending physician	39 (39.0)
Deputy chief physician	41 (41.0)
Chief physician	20 (20.0)
Patients seen, median (IQR)	
Outpatients per week	40 (20.0)
Inpatients per month	13.5 (10.0)
Rheumatologist-estimated patient characteristics (% of patients seen), median (IQR)	
Newly diagnosed outpatients	20.0 (15.0)
Follow-up patients among outpatients	80.0 (15.0)
Received drug therapy for RA among newly diagnosed outpatients	98.0 (10.0)
Prescribed regimen for newly diagnosed RA patients	
One csDMARD + NSAIDs	25.0 (22.5)
Combination of two csDMARDs	22.5 (30.0)
csDMARD alone	15.0 (20.0)
One csDMARD + glucocorticoid	10.0 (10.0)
bDMARD/tsDMARD alone	5.0 (9.0)

*bDMARD* biologic disease-modifying antirheumatic drug, *BMI* body mass index, *csDMARD* conventional synthetic disease-modifying antirheumatic drug, *HCQ* hydroxychloroquine, *IQR* interquartile range, *LEF* leflunomide, *MTX* methotrexate, *NSAIDs* nonsteroidal anti-inflammatory drugs, *RA* rheumatoid arthritis, *SASP* salazosulfapyridine, *SD* standard deviation, *tsDMARD* targeted synthetic disease-modifying antirheumatic drug

^a^Sum of *n* does not add up to 400 because patients may be prescribed two csDMARDs

^b^Includes medication for RA and comorbidities

### AEs from csDMARDs

Rheumatologist-estimated median AE rates were 15% for patients receiving MTX and LEF, and 10% for patients using SASP and hydroxychloroquine (HCQ; Table 2). On the other hand, the proportion of patients who reported experiencing AEs after treatment with MTX, LEF, SASP, and HCQ was 39.6%, 33.7%, 48.6%, and 14.6%, respectively.

**Table 2:** AEs from csDMARD use

	Participants
Rheumatologist-estimated	*N* = 100
AE rate in patients using various csDMARDs (%), median (IQR)	
MTX	15.0 (20.0)
LEF	15.0 (10.0)
SASP	10.0 (10.0)
HCQ	10.0 (5.0)
Patient-reported	*N* = 400
Experienced AEs after taking prescribed csDMARD, *n* (%)	
MTX (*n* = 202)	80 (39.6)
LEF (*n* = 172)	58 (33.7)
SASP (*n* = 35)	17 (48.6)
HCQ (*n* = 55)	8 (14.6)
Changed csDMARD, *n* (%)	113 (28.3)
Notified rheumatologist about AEs, *n* (%)^a^	116 (85.9)

*AE* adverse event, *csDMARD* conventional synthetic disease-modifying antirheumatic drug, *HCQ* hydroxychloroquine, *IQR* interquartile range, *LEF* leflunomide, *MTX* methotrexate, *SASP* salazosulfapyridine

^a^*n* = 135

As for common AEs, rheumatologists were asked either the csDMARDs-related symptoms/results were common or not, and the results of patients reported experiencing each AE were analyzed (Table 3). Similarly, nausea/vomiting, acidity/bloating and distention/loose motions, and hair loss/rash ranked the front for both rheumatologists and patients. High proportions of rheumatologists identified laboratory/imaging results including leukopenia, neutropenia, thrombocytopenia, interstitial lung disease, and liver/kidney function impairment as common AEs, while the occurrence of those AEs reported by patients was lower than expected. For MTX, canker sores were estimated as common by 51% of rheumatologists, which was higher than other csDMARDs and differed with results reported by patients.

**Table 3:** Common AEs estimated by rheumatologists and AEs reported by patients for MTX, LEF, SASP, and HCQ

	MTX		LEF		SASP		HCQ	
	Rheumatologist-estimated^a^ *N* = 100	Patient- reported^b^ *n* = 80	Rheumatologist-estimated^a^ *N* = 100	Patient-reported^b^ *n* = 58	Rheumatologist-estimated^a^ *n* = 96	Patient-reported^b^ *n* = 17	Rheumatologist-estimated^a^ *n* = 95	Patient-reported^b^ *n* = 8
Gastrointestinal symptoms, *n* (%)								
Abdominal pain	29 (29.0)	12 (15.0)	32 (32.0)	7 (12.1)	32 (33.3)	0	28 (29.5)	0
Diarrhea	30 (30.0)	13 (16.3)	34 (34.0)	12 (20.7)	20 (20.8)	1 (5.9)	14 (14.7)	0
Constipation	9 (9.0)	9 (11.3)	19 (19.0)	9 (15.5)	12 (12.5)	2 (11.8)	6 (6.3)	1 (12.5)
Nausea/vomiting	81 (81.0)	46 (57.5)	53 (53.0)	19 (32.8)	57 (59.4)	5 (29.4)	22 (23.2)	3 (37.5)
Canker sores	51 (51.0)	14 (17.5)	16 (16.0)	10 (17.2)	15 (15.6)	2 (11.8)	6 (6.3)	1 (12.5)
Acidity/bloating and distention/loose motions	37 (37.0)	34 (42.5)	37 (37.0)	25 (43.1)	26 (27.1)	6 (35.3)	19 (20.0)	3 (37.5)
Behavioral symptoms, *n* (%)								
Insomnia	21 (21.0)	16 (20.0)	15 (15.0)	11 (19.0)	12 (12.5)	2 (11.8)	22 (23.2)	1 (12.5)
Aversion to drug name, sight, and thought	7 (7.0)	2 (2.5)	2 (2.0)	1 (1.7)	8 (8.3)	0	7 (7.4)	1 (12.5)
Memory loss/difficulty concentrating	2 (2.0)	5 (6.3)	7 (7.0)	2 (3.5)	12 (12.5)	1 (5.9)	10 (10.5)	0
Anxiety/depression	10 (10.0)	6 (7.5)	11 (11.0)	6 (10.3)	17 (17.7)	0	10 (10.5)	0
Non-specific symptoms, *n* (%)								
Weakness/fatigue	28 (28.0)	16 (20.0)	27 (27.0)	16 (27.6)	23 (24.0)	7 (41.2)	19 (20.0)	1 (12.5)
Hair loss/rash	40 (40.0)	21 (26.3)	47 (47.0)	12 (20.7)	29 (30.2)	6 (35.3)	35 (36.8)	2 (25.0)
Burning in the chest/whole body feeling hot	9 (9.0)	2 (2.5)	7 (7.0)	0	10 (10.4)	1 (5.9)	12 (12.6)	0
Laboratory/imaging results, n (%)								
Leukopenia	61 (61.0)	5 (6.3)	50 (50.0)	3 (5.2)	31 (32.3)	1 (5.9)	17 (17.9)	0
Neutropenia	33 (33.0)	2 (2.5)	20 (20.0)	3 (5.2)	24 (25.0)	1 (5.9)	7 (7.4)	0
Thrombocytopenia	23 (23.0)	0	22 (22.0)	2 (3.5)	20 (20.8)	2 (11.8)	10 (10.5)	0
Interstitial lung disease	35 (35.0)	9 (11.3)	11 (11.0)	2 (3.5)	8 (8.3)	0	5 (5.3)	0
Liver/kidney function impairment	46 (46.0)	13 (16.3)	52 (52.0)	9 (15.5)	35 (36.5)	3 (17.7)	19 (20.0)	1 (12.5)

*AE* adverse event, *csDMARD* conventional synthetic disease-modifying antirheumatic drugs, *HCQ* hydroxychloroquine, *LEF* leflunomide, *MTX* methotrexate, *SASP* salazosulfapyridine

^a^Rheumatologists were asked to estimate whether each AE was common, and calculated the number and proportion of people who believed it was a common AE

^b^Patients were asked to report the AEs they once experienced and calculated the number and proportion of each AE

**Fig. 1:**
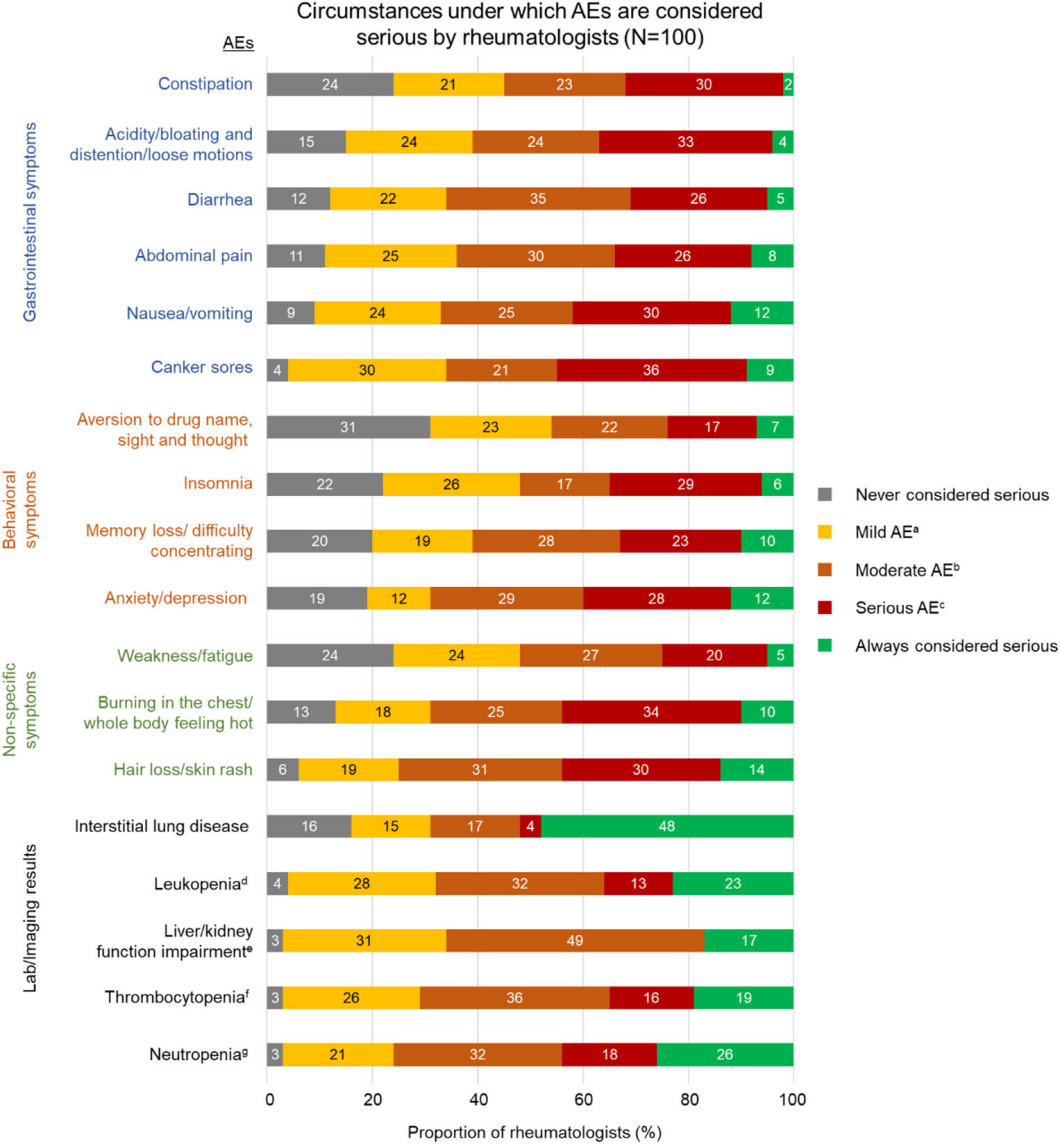
Circumstances under which the rheumatologists considered the AEs as serious (*N* = 100). AE, adverse event. ^a^Patient passively confirms the occurrence of tolerable AE. ^b^Patient actively confirms the occurrence of tolerable AE and requests adjustment of treatment. ^c^Patient actively confirms the occurrence of intolerable AE and strongly requests intervention or adjustment of treatment. ^d^Mild was defined as 3.0 × 10^9^/L, moderate as 2.0 × 10^9^/L, and severe as 1.0 × 10^9^/L. ^e^Mild was defined as 1–3 × normal value, and moderate as > 3 × normal value. Severe was not defined in the questionnaire for rheumatologists, hence not included as an option. ^f^Mild was defined as 100 × 10^9^/L, moderate as 50 × 10^9^/L, and severe as 25 × 10^9^/L. ^g^Mild was defined as 2.0 × 10^9^/L, moderate as 1.0 × 10^9^/L, and severe was defined as 0.5 × 10^9^/L

The circumstances under which rheumatologists considered an AE to be serious are presented in Fig. 1. Results indicate that rheumatologists have a higher tendency to consider AEs classified as laboratory/imaging results as serious, compared with other AEs. AEs categorized as laboratory/imaging results were associated with the highest total proportion of rheumatologists who classified them as serious AEs regardless of severity (i.e., upon occurrence), when mild (i.e., patient passively confirms the occurrence of tolerable AE), or when moderate (i.e., patient actively confirms the occurrence of tolerable AE and requests adjustment of treatment).

### Non-compliance with csDMARDs

Among 100 participating rheumatologists, the estimated mean proportion of patients who may refuse treatment with csDMARD(s) and stop or reduce the dosage of csDMARD(s) without prior consultation to be 6.7% and 12.3%, respectively. However, from 368 patients who answered the question about medication adherence, the proportions of patients that reported not strictly adhere to prescription varied from 34.5 to 54.3% among four csDMARDs (Table 4). Overall, 40.8% (150/368) of patients reported that they did not strictly adhere to their prescribed treatment; 37.5% (138/368) of patients reported that they occasionally missed a dose, 3.3% (12/368) reported frequently missing a dose, and 1.6% (6/368) of patients reduced the dose without consulting their rheumatologist (six patients chose two answers at the same time).

**Table 4:** Patient-reported compliance with MTX, LEF, SASP, and HCQ

Drug use adherence, (%)	MTX *N* = 202	LEF *N* = 172	SASP *N* = 35	HCQ *N* = 55	Total *N* = 368
Strictly adhere to doctor’s prescription	119 (58.9)	105 (61.1)	16 (45.7)	36 (65.5)	218 (59.2)
Occasionally miss a dose	74 (36.6)	64 (37.2)	18 (51.4)	19 (34.6)	138 (37.5)
Frequently miss a dose	6 (3.0)	6 (3.5)	1 (2.9)	0	12 (3.3)
Reduce the dose without consulting their doctor	5 (2.5)	1 (0.6)	2 (5.7)	1 (1.8)	6 (1.6)

The total proportion exceeded 100% due to 6 patients selecting 2 answers

*MTX* methotrexate, *LEF* leflunomide, *SASP* salazosulfapyridine, *HCQ* hydroxychloroquine

**Fig. 2:**
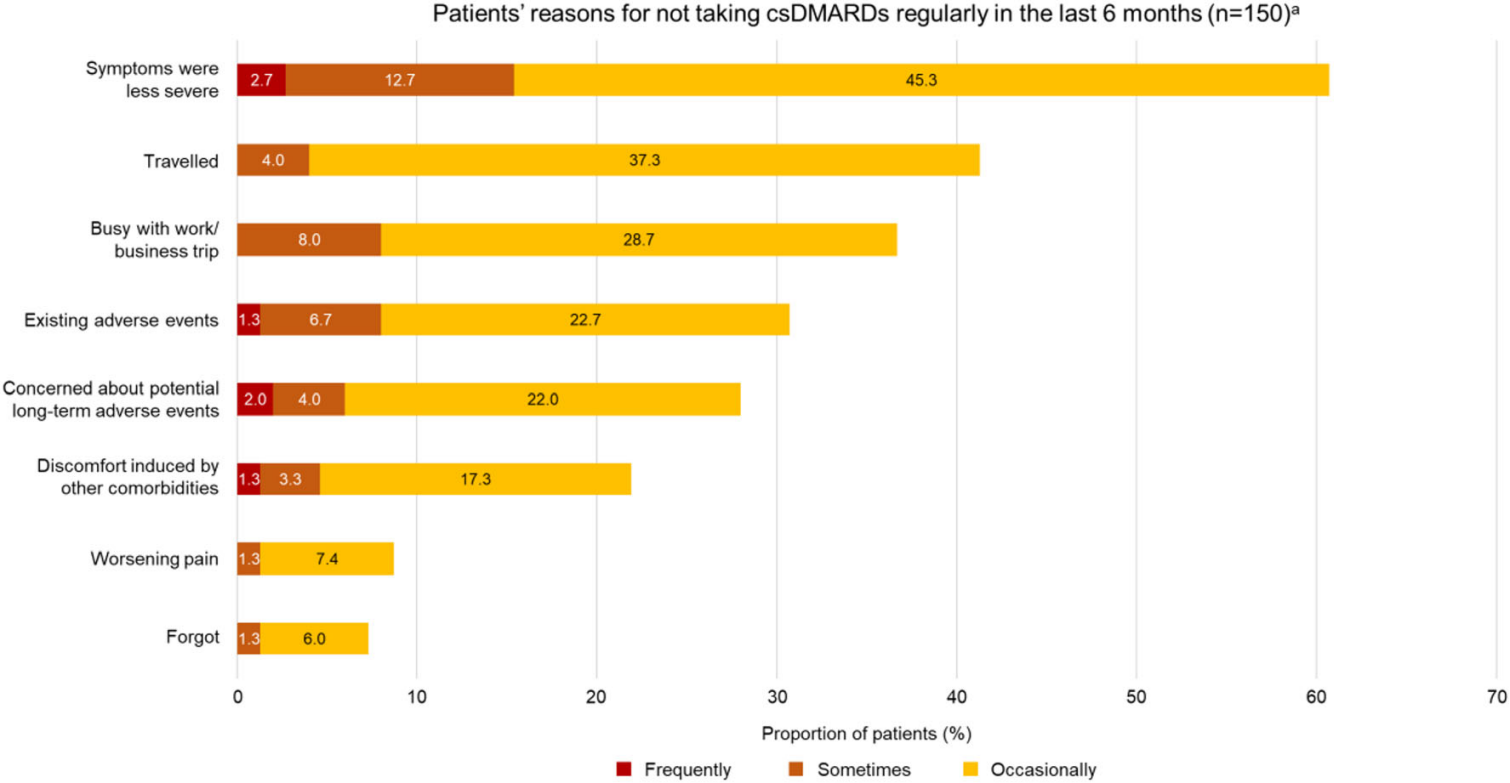
Patient-reported reasons for not taking csDMARDs regularly within the previous 6 months (*n* = 150)^a^. AE, adverse event; csDMARD, conventional synthetic disease-modifying antirheumatic drugs. ^a^Forty-nine patients reported that they did not take csDMARDs regularly in the previous 6 months because of reasons not specified in the questionnaire (frequently 2.0%; sometimes 10.2%; occasionally 71.4%)

For patients who reported not taking csDMARDs regularly, reduction in the severity of symptoms was the most common reason provided by patients (60.7%, 91/150). The next most common reasons given were travel and being busy with work/business trips (41.3%, 62/150; 36.7%, 55/150). Additionally, 30.7% (46/150) of patients who did not comply with their therapy attributed non-compliance to pre-existing AEs from treatment with csDMARD(s). Also, 28% (42/150) of patients reported non-compliance because of concerns about potential long-term AEs (Fig. 2).

### Rheumatologist-estimated ideal and actual dose of MTX and LEF

**Fig. 3:**
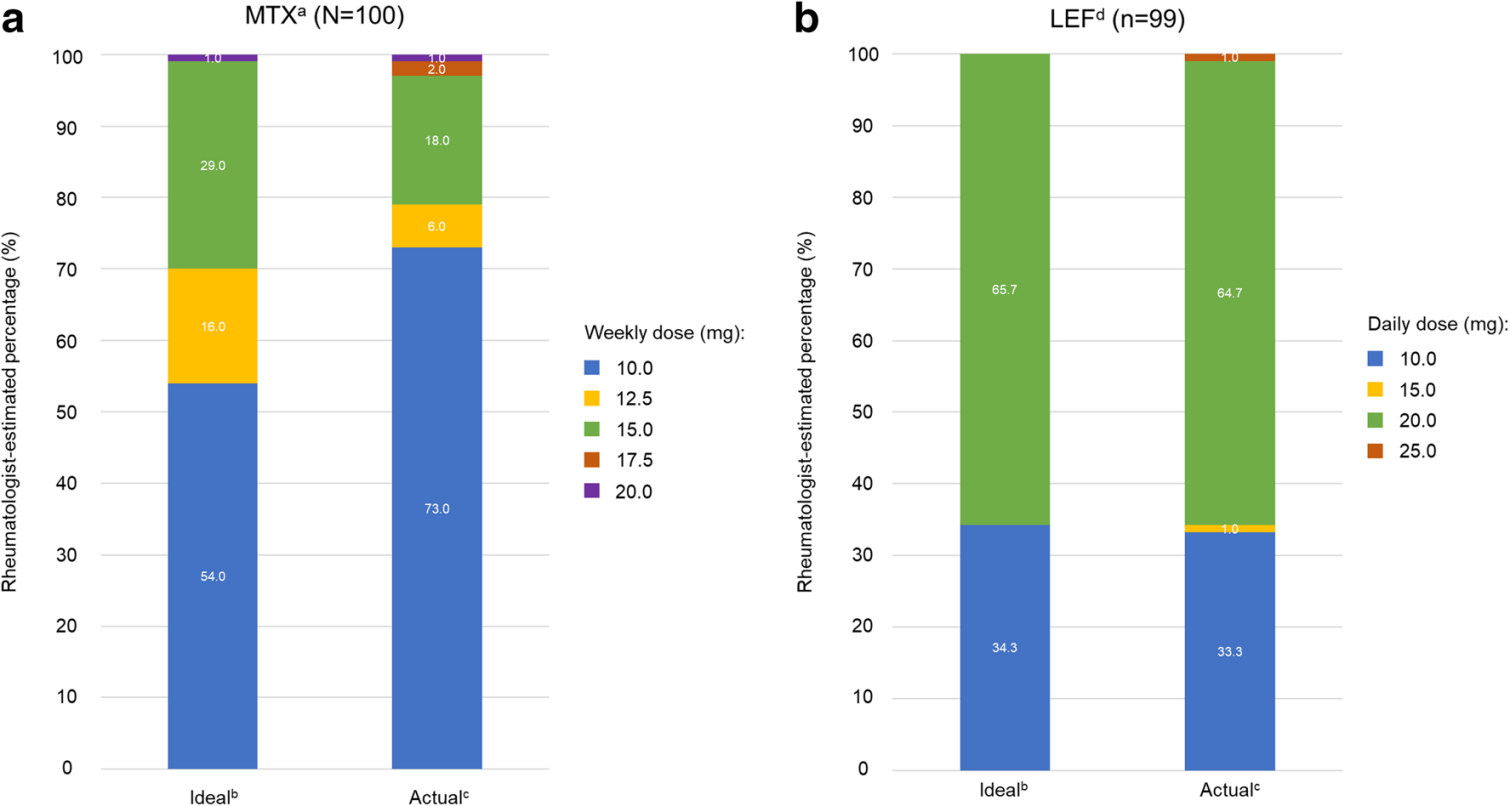
Rheumatologists’ perception of ideal versus actual dose prescribed for **a** MTX (*N* = 100) and **b** LEF (*n* = 99). LEF, leflunomide; MTX, methotrexate; RA, rheumatoid arthritis. ^a^Prescribed as either mono- or combination therapy in 66.8% of newly diagnosed patients with RA. ^b^Ideal dose is defined as the rheumatologists’ estimate of the minimum dose required to achieve treatment efficacy. ^c^Actual dose is defined as the rheumatologists’ estimate of the dose that most patients actually take. ^d^Prescribed as either mono- or combination therapy in 46.9% of newly diagnosed patients with RA

Of the rheumatologists interviewed, 54% (54/100) considered MTX 10.0 mg weekly to be the minimum dose needed to achieve efficacy (ideal dose), and 73% (73/100) considered this dose to be the most frequently prescribed MTX dose (actual dose). For LEF, 65.7% (65/99) of the rheumatologists reported 20.0 mg daily as the ideal dose, and 64.7% (64/99) reported this as the actual dose prescribed (Fig. 3).

## Discussion

RA is a significant health and economic burden in China. csDMARDs are the most widely used first-line treatment because of their affordability, efficacy, and safety [8, 24, 25]. However, compliance rates with csDMARDs (38%) in China are far from ideal [20]. In this study, we revealed gaps in perceptions of AEs and adherence related to csDMARDs between rheumatologists and their patients. Rheumatologist-estimated AE rates and types of common AEs were different from those reported by patients for all four csDMARDs. More patients reported they did not strictly adhere to their prescribed treatment than estimates of rheumatologists. We also showed that, in addition to AEs and concerns of long-term AEs, reduction in the severity of symptoms, traveling, and a busy working life/business trips are reasons why patients may not be compliant with their treatment.

The AEs for each of the four csDMARDs identified by rheumatologists differed from patient reports, suggesting that there are gaps in rheumatologists’ understanding of the occurrence and types of AEs reported by their patients. In our study, more rheumatologists but not patients regarded laboratory/imaging results, including leukopenia, neutropenia, thrombocytopenia, interstitial lung disease, and liver/kidney function impairment, as common AEs. Although, the patient-reported AEs were not evaluated by physicians and some of the AEs may not be led by csDMARDs, a possible reason for this difference in perception could be due to the communication gap. Only 86% (116/135) of patients reported that they had notified their rheumatologist about AEs. Lack of communication could lead to misestimating AE prevalence and severity among rheumatologists. In a recent global survey, 61% of patients with RA reported feeling uncomfortable about raising fears or concerns with their physicians, while 68% of physicians wished they and their patients communicated more about their RA goals and treatment [26]. Some of these patients (31%) were uncomfortable in communicating openly with physicians because of concerns that the physicians would view them as being difficult, which could influence their subsequent quality of care. Also, 14% of patients felt that they lacked knowledge or understanding of RA management. A pilot educational intervention study conducted in China revealed that the educational program on RA management not only significantly improved patients’ knowledge but also their confidence in managing their disease effectively [27]. Though many reasons contribute to the differences in perceptions of AEs, our study suggests that it is important to optimize communication between patients and their rheumatologists to improve AE recognition and reporting, and the management of RA.

Nonetheless, AEs associated with MTX use reported in this study were similar to the expected AE profile for MTX [28]. Most of the patient-reported AEs were gastrointestinal symptoms, which is consistent with a survey conducted in Australia [29]. Compared with Australian patients with RA, the prevalence rates of patient-reported nausea and skin rash were similar (23% vs ~ 28%, and 10% versus ~ 12%, respectively). The proportion of Australian patients who reported mouth ulcers (~ 25%), diarrhea (~ 20%), and stomach pain (~ 15%) was higher than that reported in our study (7%, 6.5%, and 6%, respectively). On the other hand, more patients in our study experienced vomiting than in the Australian study (23% vs ~ 7%). The AEs reported for LEF and SASP were also consistent with those reported in other multinational studies [30, 31]. However, due to the paucity in the literature of similarly designed studies, AE occurrence cannot be directly compared.

The non-compliance rate (40.8%) was within the range of those reported by other RA studies conducted in China (19.8–61.4%) [18, 19]. Non-compliance would eventually worsen disease activity, which acts against the treat-to-target strategy. Monitoring and detecting the common side effects at the early stage are essential in managing patients [23]. In this study, csDMARD treatment-related AEs were the cause of non-compliance for 30.7% of patients, and 28.0% of patients were non-compliant because they were concerned about potential long-term AEs. However, it is notable that multiple other factors may result in non-compliance, including a reduction in the severity of symptoms, travel, and busy working life/business trips. In addition, most newly diagnosed patients were prescribed two types of RA drugs (62.5%), and patients with RA took a median of three types of drugs and six tablets a day. Patients who took more RA medications had a 1.7-fold higher likelihood of non-compliance with their prescribed treatment [32]. These findings show that AEs and concerns for long-term AEs are important but are not the only reasons why patients may not strictly follow their prescribed treatment. Complex real-world situations should be considered that may lead to non-compliance.

Rheumatologists’ estimation of ideal dosage is the same as the recommended dosage by EULAR for LEF, but not for MTX [8, 33]. EULAR recommends a rapid escalation of MTX dosage to 20–25 mg/week within 4–6 weeks [7]. However, this recommended dosage does not apply to Asian patients [8]. In China and Japan, the dose of MTX should not exceed 20 mg/week and 16 mg/week, respectively [34, 35]. This difference in dosage between Western and Asian populations could possibly be due to the lower body weight and different pharmacogenetic characteristics of the Asian population [7, 36]. Nonetheless, EULAR guidelines recommend the timely adjustment of therapy if there is no improvement in the patient’s condition, or if the treatment target has not been reached [7]. In patients who have failed phase I treatment and present poor prognostic factors, such as failure of ≥ 2 csDMARDs, a bDMARD or tsDMARD should be added for phase II therapy [7].

The main limitation of this cross-sectional study is that sampling is convenient, not random, which results in selection bias and reduced reliability of the derived conclusions. In this study, the female to male ratio of patients with RA was 2:1, which deviates from the 6:1 ratio reported in other studies conducted in China [18, 20]. Second, the AEs reported by patients were not all evaluated by physicians, which affected the accuracy and may introduce recall bias. Even though AEs that are estimated by rheumatologists and AEs that patients reported in the survey cannot be compared directly, the trends highlighted a gap between the rheumatologists’ perception of clinically relevant AEs and the AEs that patients believe to result from csDMARD use. However, there were strengths of this study; our study recruited patients from 13 cities across China and, thus, may provide a more holistic representation of the Chinese patient population [18, 20]. We conducted questionnaires for both physicians and patients (Online Resource 1 and 2). Surveys investigating intolerance and/or compliance in patients with RA are often focused on patients only [18, 20, 23]. With the additional insights from rheumatologists, this study was able to identify the differences in perceptions of patients versus rheumatologists.

Our results revealed differences in perceptions between patients and rheumatologists in terms of csDMARDs-related AEs and adherence. Patients were less compliant when the severity of RA symptoms was reduced, if they had travel commitments, were busy with work, had pre-existing AEs, and if they had concerns about potential long-term AEs. These findings indicate the importance of adequate doctor-patient communication and consideration of multiple scenarios of non-adherence in the management of RA patients.

## Electronic supplementary material

ESM 1 (PDF 382 kb)

ESM 2 (PDF 396 kb)

## Data Availability

Qualified researchers may request access to individual patient level data through the clinical study data request platform (https://vivli.org/). Further details on Roche’s criteria for eligible studies are available here (https://vivli.org/members/ourmembers/). For further details on Roche’s Global Policy on the Sharing of Clinical Information and how to request access to related clinical study documents, see here (https://www.roche.com/research_and_development/who_we_are_how_we_work/clinical_trials/our_commitment_to_data_sharing.htm).
