# Supplementary material for: Investigating the safety and compliance of using csDMARDs in rheumatoid arthritis treatment through face-to-face interviews: a cross-sectional study in China
Source: Clin Rheumatol. 2020 Oct 15;40(5):1789–98. doi: 10.1007/s10067-020-05458-w (PMC8102276; doi:10.1007/s10067-020-05458-w)
Supplement: Supplementary file 1 — (PDF 382 kb) [file 10067_2020_5458_MOESM1_ESM.pdf]

Online Resource 1 – Patient questionnaire

Journal: Clinical Rheumatology

Title: Investigating the safety and compliance of using csDMARDs in rheumatoid arthritis treatment through face-to-face interviews: a cross-sectional study in China

Authors: Jiaying Sun, Siming Dai, Ling Zhang, Yajing Feng, Xin Yu, Zhiyi Zhang*

*** Corresponding author:**

Zhiyi Zhang

Department of Rheumatology and Immunology, First Affiliated Hospital of Harbin Medical University, Harbin 150086, Heilongjiang, China

Email: zhangzhiyi2014@163.com

Standard wording for interviewers before the investigational interview in the recruitment period

Interviewer:

We are going to have an investigational interview with you. We need to inform our client of the adverse events and product technical complaints that we learn from the investigational interview. Although this is an investigational interview, we will keep what you said confidential, but if you put forward one or more adverse events or product technical complaints in the interview, we will need to report on it.

In this case, we will ask for your opinion if you are willing to give up your right to request confidentiality of adverse events or product technical complaints provided by the *Code of Conduct for Market Investigation*. We will still keep the other contents you said during the interview confidential.

On this basis, are you willing to participate in this interview?

<table>
<tr><td rowspan="3">Respondent</td><td>Name</td><td></td><td>Tel</td><td colspan="3"></td></tr>
<tr><td>Hospital</td><td></td><td>Department</td><td colspan="3"></td></tr>
<tr><td>Date of interview</td><td>MM-DD</td><td>Interview time</td><td colspan="3">From HH:MM to HH:MM,
____minute(s) in total</td></tr>
<tr><td rowspan="2">Staff</td><td>Name of interviewer</td><td></td><td>Name of first reviewer</td><td></td><td>Time of first review</td><td></td></tr>
<tr><td>Name of coder</td><td></td><td>Name of entry clerk</td><td></td><td>Supervision</td><td></td></tr>
</table>

<u>Self-introduction:</u>

My name is _________, and I'm an interviewer from Adelphi FocusRx. We are conducting an investigation on rheumatoid arthritis. We sincerely invite you to participate in this interview. This interview will last for about 45 minutes. All information you provide will be kept confidential. Please speak freely. Thank you!

The interviewer promises:

I am absolutely aware of the impact of my attitude on the investigation results.

I ensure that all the information in this questionnaire is completed by me in accordance with the interview procedure and is absolutely true. If one fraud is found, all the documents will be invalidated and I will compensate the company for the loss.

Signature of interviewer:

S-screening questionnaire

S1. [Circled by the interviewer] City of Respondent?

**First-tier city**	Beijing	1	Shanghai	2	Guangzhou	3
**Second-tier city**	Xi'an	4	Wuhan	5	Fuzhou	6
	Nanjing	7	Hangzhou	8	Chengdu	9
	Shenyang	10	Ji'nan	11	Zhengzhou	12
	Changsha	13				

S2. [Single answer] How long have you been **diagnosed with rheumatoid arthritis**?

≥3 months	1	Continue
<3 months	2	Thank and end

S3. [Single answer] Have you ever used csDMARD drug such as **methotrexate, leflunomide and sulfasalazine** to treat rheumatoid arthritis? ***(If the patient is unable to judge, the interviewer shall confirm whether the drug taken is csDMARD drug according to the card and the medicine box brought by the patient)***

Under use	1	Answer S4
Used before and stopped	2	Answers S5 and S6
Never used	3	Thank and end

S4. [Single answer] How long have you been using **csDMARD drug** to treat rheumatoid arthritis?

≥3 months	1	Up to the criteria, begin to answer the main questionnaire
<3 months	2	Thank and end

S5. [Single answer] How long have you stopped using **csDMARD drug**?

≥3 months	1	Thank and end
<3 months	2	Continue

S6. [Single answer] How long have you been using **csDMARD drug** continuously before stopping use?

≥3 months	1	Up to the criteria, begin to answer the main questionnaire
<3 months	2	Thank and end

Main Questionnaire

Part I: Basic Information of Rheumatoid Arthritis Patients

Q1. [Single answer] What is your gender?

No.	Gender	Option
1	Male	□
2	Female	□

Q2. [Complete] What are your age, height and body weight?

No.	Item	Complete
1	Age	__________years old
2	Height	_________cm
3	Body weight	_________kg

Q3. [Single answer] What is your educational background?

No.	Degree of education	Option
1	High school or below	□
2	College or bachelor	□
3	Master or above	□
4	Others, please specify__________	□

Q4. [Single answer] What is your **monthly family income**?

No.	Monthly family income (Yuan)	Option
1	<5000	□
2	5000 ~ 10000	□
3	10001 ~ 20000	□
4	>20001	□

Q5. [Single answer] What is your current **type of health insurance**?

No.	Health insurance type	Option
1	Basic medical insurance for urban workers	□
2	Basic medical insurance for urban residents	□
3	New rural cooperative medical-care system	□
4	Commercial health insurance	□
5	Others, please specify__________	□

Q6. [Single answer] What is your current working status?

No.	Working status	Option
1	Full-time	□
2	Part-time	□
3	Unemployed	□
4	Student	□
5	Retired	□
6	Others, please specify__________	□

Q7. [Single answer] What is your current residence status?

No.	Residence status	Option
1	Live alone	□
2	Live with family (spouse, children)	□
3	Live in a third-party institution (such as a nursing home)	□
4	Others, please specify__________	□

Q8. [Single answer] Under normal circumstances, how long does it take to travel from your place of residence to the hospital?

No.	Residence status	Option
1	<30 minutes	□
2	30~60 minutes	□
3	61~120 minutes	□
4	>120 minutes	□

Q9. [Multiple answers] Besides rheumatoid arthritis, what other diseases do you have?

No.	Other diseases	Option
1	Hypertension	□
2	Coronary heart disease	□
3	Diabetes mellitus	□
4	Hyperlipidaemia	□
5	Respiratory disease (such as interstitial lung disease, etc.)	□
6	Stroke	□
7	Tumor	□
8	Chronic liver disease	□
9	Chronic kidney disease	□
10	Other rheumatic diseases	□
11	Others, please specify__________	□

Q10. [Complete] How many different drugs do you need to take every day to treat all diseases? How many tablets do you need to take on average every day?

No.	Item	Complete
1	Types of drugs taken every day	__________types
2	Average number of tablets taken per day	_________tablets
		Q10.2≥Q10.1

Part II: Medical information on rheumatoid arthritis

Q11. [Complete] When did you first have symptoms of rheumatoid arthritis?

Time when rheumatoid arthritis symptoms first appeared	____________(Year)____________(Month)

Q12. [Complete] When was the first time you went to the hospital for rheumatoid arthritis?

When did you go to the hospital for the first time due to rheumatoid arthritis?	____________(Year)____________(Month)

Q13. [Complete] When were you **diagnosed** with rheumatoid arthritis?

Time for diagnosis of rheumatoid arthritis	____________(Year)____________(Month)

Q14. [Single answer] Did you start drug therapy immediately after you were diagnosed with rheumatoid arthritis?

No.	Option
1	□ Yes
2	□ No, drug therapy began after ___________months of diagnosis.

Part III: Current Drug Therapy for Rheumatoid Arthritis Patients

Q15. [Complete] What drugs are you using to **treat rheumatoid arthritis**? Strength? Route of administration? Frequency of administration? Dose?

Category	1 Current therapeutic drugs	2 Strength	3 Route of administration	4 Frequency of administration	5 Dose
csDMARD	□ Methotrexate	___mg/ tablet & vial	□ Oral<br>□ Injection	___times/<br>□ day □ week	___tablet/vial
	□ Leflunomide	___mg/ tablet & vial	□ Oral<br>□ Injection	___times/<br>□ day □ week	___tablet/vial
	□ Sulfasalazine	___mg/ tablet & vial	□ Oral<br>□ Injection	___times/<br>□ day □ week	___tablet/vial
	□ Hydroxychloroquine	___mg/ tablet & vial	□ Oral<br>□ Injection	___times/<br>□ day □ week	___tablet/vial
	□ Others______	___mg/ tablet & vial	□ Oral<br>□ Injection	___times/<br>□ day □ week	___tablet/vial
b/tsDMARD	□ Etanercept (Enbrel)	___mg/ tablet & vial	□ Oral<br>□ Injection	___times/<br>□ day □ week	___tablet/vial
	□ Etanercept (Etanar)	___mg/ tablet & vial	□ Oral<br>□ Injection	___times/<br>□ day □ week	___tablet/vial
	□ Rituximab (MabThera)	___mg/ tablet & vial	□ Oral<br>□ Injection	___times/<br>□ day □ week	___tablet/vial
	□ Infliximab (Remicade)	___mg/ tablet & vial	□ Oral<br>□ Injection	___times/<br>□ day □ week	___tablet/vial
	□ Adalimumab (Humira)	___mg/ tablet & vial	□ Oral<br>□ Injection	___times/<br>□ day □ week	___tablet/vial
	□ Tocilizumab (Actemera)	___mg/ tablet & vial	□ Oral<br>□ Injection	___times/<br>□ day □ week	___tablet/vial
	□ Tofacitinib (Xeljanz)	___mg/ tablet & vial	□ Oral<br>□ Injection	___times/<br>□ day □ week	___tablet/vial
	□ Others: ______	___mg/ tablet & vial	□ Oral<br>□ Injection	___times/<br>□ day □ week	___tablet/vial
□ Others: ________________________		___mg/ tablet & vial	□ Oral<br>□ Injection	___times/<br>□ day □ week	___tablet/vial
□ Others: ________________________		___mg/ tablet & vial	□ Oral<br>□ Injection	___times/<br>□ day □ week	___tablet/vial

Q16. [Single answer] Can you accept the number of drugs currently taken to treat rheumatoid arthritis?

No.	Number of drugs taken	Option
1	There are too many drugs, I hope they can be reduced.	□
2	There are too many drugs, but they are tolerable.	□
3	The number of drugs is just right, not too much.	□
4	Others: ___________________	□

Q17. [Single answer] How do you feel about the effect after using the above therapeutic regimen?

No.	Effect evaluation	Option
1	The effect is very good.	□
2	The effect is good.	□
3	The effect is modest.	□
4	The effect is not good.	□
5	It has no effect at all.	□

Q18. [Complete] How do you define “good effect”? Please rank the following criteria on your importance scale of 1 to 7. The higher it ranks, the more important it is.

No.	“Good effect” criteria	Option
1	After administration, the symptoms of joint swelling and pain can be quickly relieved.	Rank___
2	After administration, the symptoms can be completely relieved without affecting normal life.	Rank___
3	After administration, the symptoms relieving effect can last until the next dose.	Rank___
4	The hospital examination indicators reach the normal value.	Rank___
5	There is no obvious side effect after taking the medicine.	Rank___
6	The doctor tells me that the therapy is effective.	Rank___
7	Able to improve long-term prognosis and prevent joint deformity.	Rank___
8	Others, please specify__________	Rank___

Q19. [Transverse single answer] After using the current therapeutic regimen, do you think it meets the following criteria?

No.	“Good effect” criteria	Option
1	The symptoms can be relieved quickly after taking the medicine.	□ Yes □ No
2	After administration, the symptoms can be completely relieved without affecting normal life.	□ Yes □ No
3	After administration, the symptoms relieving effect can last until the next dose.	□ Yes □ No
4	The hospital examination indicators reach the normal value.	□ Yes □ No
5	There is no obvious side effect after taking the medicine.	□ Yes □ No
6	The doctor tells me that the therapy is effective.	□ Yes □ No
7	I believe that the therapy can improve long-term prognosis and prevent joint deformity.	□ Yes □ No
8	Others, please specify__________	□ Yes □ No

Q20. [Single answer] How do you evaluate your health compared to a year ago?

No.	Health assessment	Option
1	Much better	□
2	A little better	□
3	Almost the same	□
4	Worse	□
5	Much worse	□
6	Others, please specify__________	□

Q21. [Single answer] In order to achieve the desired therapeutic effect of rheumatoid arthritis, how much do you think is appropriate to spend each month?

No.	Cost of treatment	Option
1	<1,000 yuan	□
2	1,000 ~ 1,999 yuan	□
3	2,000 ~ 4,999 yuan	□
4	5,000 ~ 9,999 yuan	□
5	≥10,000 yuan	□

[Answer Q22 for patients who select csDMARD drug in Q15, otherwise skip to Q32]

Q22. [Single answer] Taking **csDMARD drug** (the type selected in Q15) has become your daily (LEF) / weekly (MTX) routine?

No.	Degree of consent	Option
1	Totally agree	□
2	Agree	□
3	Not quite agree	□
4	Totally disagree	□

Q23. [Single answer] It is easy for you to take **csDMARD drug** (the type selected in Q15).

No.	Degree of consent	Option
1	Totally agree	□
2	Agree	□
3	Not quite agree	□
4	Totally disagree	□

Q24. [Multiple answers] As for **csDMARD drug** you are using (the type selected in Q15), do you take them on time in strict accordance with the doctor's advice?

No.	Drug use compliance	Option
1	Take medicine on time in strict accordance with the doctor's advice.	□
2	Occasionally miss a dose.	□
3	Frequently miss a dose.	□
4	Reduce the dose without the doctor’s advice.	□
5	Add the dose without the doctor’s advice.	□

[If Q24=2 or 3, answer Q25, otherwise skip to Q26]

Q25. [Transverse single answer] For **csDMARD drug** you are using (the type selected in Q15), what is the frequency of irregular administration **in the past six months** due to the following reasons?

No.	Reason for irregular administration	Frequency of missing a dose due to this reason
1	worsening pain	□ Often □ Sometimes □ Occasionally □ Never
2	Discomfort induced by other disease	□ Often □ Sometimes □ Occasionally □ Never
3	Relief of symptoms	□ Often □ Sometimes □ Occasionally □ Never
4	Consideration of possible long-term side effects	□ Often □ Sometimes □ Occasionally □ Never
5	Existing side effects	□ Often □ Sometimes □ Occasionally □ Never
6	Busy work or business trip	□ Often □ Sometimes □ Occasionally □ Never
7	Travel	□ Often □ Sometimes □ Occasionally □ Never
8	Forget simply because of poor memory.	□ Often □ Sometimes □ Occasionally □ Never
9	Others________	□ Often □ Sometimes □ Occasionally □ Never

Q26. [Single answer] For the **csDMARD drug** you are using to treat rheumatoid arthritis (the type selected in Q15), do you have any side effects after administration?

No.	Is there any side effect?	Option
1	Yes	□
2	No	□

[If Q26=1, answer Q27, otherwise skip to Q32]

Q27. [Longitudinal multiple answers] **[For csDMARD drug selected in Q15]** What are the side effects after using csDMARD drug? How about your tolerance of these side effects, please rank them, with 1 indicating "least tolerable".

Category of side effect		Side effects	Methotrexate		Leflunomide		Sulfasalazine		Hydroxy-chloroquine		Others_____	
Patient's chief complaints	Gastrointestinal reaction	Abdominal pain	□	Rank_	□	Rank_	□	Rank_	□	Rank_	□	Rank_
		Diarrhoea	□	Rank_	□	Rank_	□	Rank_	□	Rank_	□	Rank_
		Constipation	□	Rank_	□	Rank_	□	Rank_	□	Rank_	□	Rank_
		Nausea, vomiting	□	Rank_	□	Rank_	□	Rank_	□	Rank_	□	Rank_
		Dental ulcer	□	Rank_	□	Rank_	□	Rank_	□	Rank_	□	Rank_
		Acid regurgitation, abdominal distension and insufficient gastric motility	□	Rank_	□	Rank_	□	Rank_	□	Rank_	□	Rank_
	Behavioral expression	Insomnia	□	Rank_	□	Rank_	□	Rank_	□	Rank_	□	Rank_
		Disgusted with the name/shape of the drug	□	Rank_	□	Rank_	□	Rank_	□	Rank_	□	Rank_
		Memory loss, difficulty in concentration	□	Rank_	□	Rank_	□	Rank_	□	Rank_	□	Rank_
		Anxiety, depression	□	Rank_	□	Rank_	□	Rank_	□	Rank_	□	Rank_
	Nonspecific manifestation	Weakness, fatigue	□	Rank_	□	Rank_	□	Rank_	□	Rank_	□	Rank_
		Hair loss, rash	□	Rank_	□	Rank_	□	Rank_	□	Rank_	□	Rank_
		Dryness-heat and chest burning	□	Rank_	□	Rank_	□	Rank_	□	Rank_	□	Rank_
Laboratory results		Leukopenia	□	Rank_	□	Rank_	□	Rank_	□	Rank_	□	Rank_
		neutropenia	□	Rank_	□	Rank_	□	Rank_	□	Rank_	□	Rank_
		thrombocytopenia	□	Rank_	□	Rank_	□	Rank_	□	Rank_	□	Rank_
		Interstitial lung disease	□	Rank_	□	Rank_	□	Rank_	□	Rank_	□	Rank_
		Impairment of liver and kidney functions (such as hematuresis)	□	Rank_	□	Rank_	□	Rank_	□	Rank_	□	Rank_
Others________			□	Rank_	□	Rank_	□	Rank_	□	Rank_	□	Rank_

Q28. [Single answer] Have you complained or reflected to the doctor about the side effects after using csDMARD drug?

No.	Have you complained or reflected to the doctor?	Option
1	Yes	□
2	No	□

[If Q28=1, answer Q29, otherwise skip to Q31]

Q29. [Longitudinal multiple answers] **[csDMARD drug selected in Q15]** What side effects have you complained or reflected to the doctor?

Category of side effect		Side effects	Methotrexate	Leflunomide	Sulfasalazine	Hydroxychloroquine	Others___
Patient's chief complaints	Gastrointestinal reaction	Abdominal pain	□	□	□	□	□
		Diarrhoea	□	□	□	□	□
		Constipation	□	□	□	□	□
		Nausea, vomiting	□	□	□	□	□
		Dental ulcer	□	□	□	□	□
		Acid regurgitation, abdominal distension and insufficient gastric motility	□	□	□	□	□
	Behavioral expression	Insomnia	□	□	□	□	□
		Disgusted with the name/shape of the drug	□	□	□	□	□
		Memory loss, difficulty in concentration	□	□	□	□	□
		Anxiety, depression	□	□	□	□	□
	Nonspecific manifestation	Weakness, fatigue	□	□	□	□	□
		Hair loss, rash	□	□	□	□	□
		Dryness-heat and chest burning	□	□	□	□	□
Laboratory results		Leukopenia	□	□	□	□	□
		neutropenia	□	□	□	□	□
		thrombocytopenia	□	□	□	□	□
		Interstitial lung disease	□	□	□	□	□
		Impairment of liver and kidney functions (such as hematuresis)	□	□	□	□	□
Others________			□	□	□	□	□

Q30. [Single answer] After complaining or reflecting the side effects to the doctor, do you require the doctor to adjust the therapeutic regimen?

No.	The patient's advice after reporting side effects to the doctor	Option
1	Ask the doctor to reduce the dose but not change the drug.	□
2	Ask the doctor to change the drug.	□
3	Only complain or reflect the side effects to the doctor, without proposing other requirements.	□

Q31. [Single answer] Have you changed the therapy by yourself for the side effects that occurred after using csDMARD drug?

No.	Actions taken without the doctor's advice	Option
1	Withdraw the drug	□
2	Reduce the dose	□
3	Irregular medication (e.g. no medication if side effects are serious)	□
4	Change the drug	□
5	Others_______	□

Q32. [Single answer] What's the frequency of your follow-up visit/re-examination in the hospital?

No.	Frequency	Option
1	Once every two weeks	□
2	Once a month	□
3	Every three months	□
4	Every half year	□
5	When symptoms such as joint pain recur	□
6	When other complications occur	□
7	When the patient wants to adjust the therapeutic regimen as the condition is relieved	
8	Others: __________________	□

Part IV: Preceding Drug Therapy for Rheumatoid Arthritis Patients

Q33. [Single answer] Have you changed csDMARD drug before?

No.	Have you ever changed csDMARD drug	Option
1	Yes	□
2	No	□

[If Q33=1, answer Q34, otherwise skip to Q37]

Q34. [Complete] What other csDMARD drugs have you used to treat rheumatoid arthritis ***(multiple choices)***? Strength? Route of administration? Frequency of administration?

Category	1 Previous drug	2 Strength	3 Route of administration	4 Frequency of administration	5 Dose
csDMARD	□ Methotrexate	___mg/ tablet & vial	□ Oral □ Injection	___times/ □ day □ week	___tablet/vial
	□ Leflunomide	___mg/ tablet & vial	□ Oral □ Injection	___times/ □ day □ week	___tablet/vial
	□ Sulfasalazine	___mg/ tablet & vial	□ Oral □ Injection	___times/ □ day □ week	___tablet/vial
	□ Hydroxychloroquine	___mg/ tablet & vial	□ Oral □ Injection	___times/ □ day □ week	___tablet/vial
	□ Others______	___mg/ tablet & vial	□ Oral □ Injection	___times/ □ day □ week	___tablet/vial

Q35. [Longitudinal multiple answers] **[For csDMARD drug selected in Q34]** What is the reason for changing the therapeutic regimen?

No.	Primary reason	Methotrexate	Leflunomide	Sulfasalazine	Hydroxychloroquine	Others__
1	The disease has been brought under control.	□	□	□	□	□
2	The disease has not been effectively controlled.	□	□	□	□	□
3	It is inconvenient to use the drug.	□	□	□	□	□
4	Heavy economic burden	□	□	□	□	□
5	Serious side effects	□	□	□	□	□
6	The doctor suggested a replacement.	□	□	□	□	□
7	The patient proposed to change the drug, and the doctor agreed.	□	□	□	□	□
8	Others, please specify__________	□	□	□	□	□

[If Q35=5, answer Q36, otherwise skip to Q37]

Q36. [Longitudinal multiple answers] **[For csDMARD drug with 5 selected in Q35]** What are the main side effects that lead to the change of therapeutic regimen before?

Category of side effect		Side effects	Methotrexate	Leflunomide	Sulfasalazine	Hydroxychloroquine	Others__
Patient's chief complaints	Gastrointestinal reaction	Abdominal pain	□	□	□	□	□
		Diarrhoea	□	□	□	□	□
		Constipation	□	□	□	□	□
		Nausea, vomiting	□	□	□	□	□
		Dental ulcer	□	□	□	□	□
		Acid regurgitation, abdominal distension and insufficient gastric motility	□	□	□	□	□
	Behavioral expression	Insomnia	□	□	□	□	□
		Disgusted with the name/shape of the drug	□	□	□	□	□
		Memory loss, difficulty in concentration	□	□	□	□	□
		Anxiety, depression	□	□	□	□	□
	Nonspecific manifestation	Weakness, fatigue	□	□	□	□	□
		Hair loss, rash	□	□	□	□	□
		Dryness-heat and chest burning	□	□	□	□	□
Laboratory results		Leukopenia	□	□	□	□	□
		neutropenia	□	□	□	□	□
		thrombocytopenia	□	□	□	□	□
		Interstitial lung disease	□	□	□	□	□
		Impairment of liver and kidney functions (such as hematuresis)	□	□	□	□	□
Others________			□	□	□	□	□

Part V: Cognition and Unmet Needs of Rheumatoid Arthritis Patients

Q37. [Transverse single answer] Have you heard of the following concepts or information about rheumatoid arthritis?

Q38. [Transverse single answer] **[For "Yes" answer in Q37]** How well do you know these concepts or information?

No.	Concepts or information related to rheumatoid arthritis	Q37	Q38
1	Rheumatoid arthritis treatment requires treat-to-target	□ Yes<br>□ No	□ Heard of it, but know nothing about the details<br>□ Heard of it, know a little bit of the information<br>□ Know about it<br>□ Very clear about the details
2	Rheumatoid arthritis is a chronic disease and needs regular monitoring and follow-up	□ Yes<br>□ No	□ Heard of it, but know nothing about the details<br>□ Heard of it, know a little bit of the information<br>□ Know about it<br>□ Very clear about the details
3	Rheumatoid arthritis requires simultaneous attention to joint and systemic symptoms.	□ Yes<br>□ No	□ Heard of it, but know nothing about the details<br>□ Heard of it, know a little bit of the information<br>□ Know about it<br>□ Very clear about the details
4	Long-term prognosis and disability of rheumatoid arthritis	□ Yes<br>□ No	□ Heard of it, but know nothing about the details<br>□ Heard of it, know a little bit of the information<br>□ Know about it<br>□ Very clear about the details
5	Biological/targeted therapy for rheumatoid arthritis	□ Yes<br>□ No	□ Heard of it, but know nothing about the details<br>□ Heard of it, know a little bit of the information<br>□ Know about it<br>□ Very clear about the details

Q39. [Transverse multiple answers] **[For "Yes" answer in Q37]** From which channels do you obtain those information?

No.	Concepts or information related to rheumatoid arthritis	Doctor	Nurse	Family/ colleague	Wardmate	Patient Education Association	Internet	WeChat/ Microblog	Professional books and magazines	Others__
1	Rheumatoid arthritis treatment requires treat-to-target	□	□	□	□	□	□	□	□	□
2	Rheumatoid arthritis is a chronic disease and needs regular monitoring and follow-up	□	□	□	□	□	□	□	□	□
3	Rheumatoid arthritis requires simultaneous attention to joint and systemic symptoms.	□	□	□	□	□	□	□	□	□
4	Prognosis and disability of rheumatoid arthritis	□	□	□	□	□	□	□	□	□
5	Biological/targeted therapy for rheumatoid arthritis	□	□	□	□	□	□	□	□	□

Q40. [Multiple answers] What are your expectations or unmet needs for drug therapy for rheumatoid arthritis? ***(Select up to 3 items)***

No.	Expectations or unmet needs	Option
1	Quickly relieve joint swelling and pain	□
2	Lasting curative effect	□
3	Control or prevent other complications	□
4	Help to improve the life quality	□
5	Protect joints and prevent long-term disability	□
6	Convenient use	□
7	Lower frequency of administration	□
8	Less side effects	□
9	Lower treatment costs	□
10	Others, please specify__________	□

Q41. [Single answer] Do you know anything about daily nursing, diet control and moderate exercise required for rheumatoid arthritis?

No.	Know	Option
1	Yes	□
2	No	□

Q42. [Multiple answers] What do you want to know about rheumatoid arthritis?

No.	Item	Option
1	Therapeutic drug	□
2	Daily nursing	□
3	Dietary advice	□
4	Scientific knowledge about the disease	□
5	Online expert consultation	□
6	Others, please specify__________	□

Q43. [Multiple answers] From which channel do you want to know most about rheumatoid arthritis? ***(Select up to 3 items)***

No.	Channel	Option
1	Doctor	□
2	Nurse	□
3	Family/colleague	□
4	Wardmate	□
5	Patient Education Association	□
6	Internet	□
7	WeChat/Microblog	□
8	Professional books and magazines	□
9	Others, please specify__________	□
