# Supplementary material for: Investigating the safety and compliance of using csDMARDs in rheumatoid arthritis treatment through face-to-face interviews: a cross-sectional study in China
Source: Clin Rheumatol. 2020 Oct 15;40(5):1789–98. doi: 10.1007/s10067-020-05458-w (PMC8102276; doi:10.1007/s10067-020-05458-w)
Supplement: Supplementary file 2 — (PDF 396 kb) [file 10067_2020_5458_MOESM2_ESM.pdf]

Online Resource 2 – Rheumatologist questionnaire

Journal: Clinical Rheumatology

Title: Investigating the safety and compliance of using csDMARDs in rheumatoid arthritis treatment through face-to-face interviews: a cross-sectional study in China

Authors: Jiaying Sun, Siming Dai, Ling Zhang, Yajing Feng, Xin Yu, Zhiyi Zhang*

*** Corresponding author:**

Zhiyi Zhang

Department of Rheumatology and Immunology, First Affiliated Hospital of Harbin Medical University, Harbin 150086, Heilongjiang, China

Email: zhangzhiyi2014@163.com

Standard wording for interviewers before the investigational interview in the recruitment period

Interviewer:

We are going to have an investigational interview with you. We need to inform our client of the adverse events and product technical complaints that we learn from the investigational interview. Although this is an investigational interview, we will keep what you said confidential, but if you put forward one or more adverse events or product technical complaints in the interview, we will need to report on it.

In this case, we will ask for your opinion if you are willing to give up your right to request confidentiality of adverse events or product technical complaints provided by the *Code of Conduct for Market Investigation*. We will still keep the other contents you said during the interview confidential.

On this basis, are you willing to participate in this interview?

<table>
<tr><td rowspan="3">Respondent</td><td>Name</td><td></td><td>Tel</td><td colspan="3"></td></tr>
<tr><td>Hospital</td><td></td><td>Department</td><td colspan="3"></td></tr>
<tr><td>Date of interview</td><td>MM-DD</td><td>Interview time</td><td colspan="3">From HH:MM to HH:MM, ___minute(s) in total</td></tr>
<tr><td rowspan="2">Staff</td><td>Name of interviewer</td><td></td><td>Name of first reviewer</td><td></td><td>Time of first review</td><td></td></tr>
<tr><td>Name of coder</td><td></td><td>Name of entry clerk</td><td></td><td>Supervision</td><td></td></tr>
</table>

<u>Self-introduction:</u>

My name is _________, and I'm an interviewer from Adelphi FocusRx. We are conducting an investigation on rheumatoid arthritis. In view of your rich experience in this field, we sincerely invite you to participate in this interview. This interview will last for about 45 minutes. All information you provide will be kept confidential. Please speak freely. Thank you!

The interviewer promises:

I am absolutely aware of the impact of my attitude on the investigation results.

I ensure that all the information in this questionnaire is completed by me in accordance with the interview procedure and is absolutely true. If one fraud is found, all the documents will be invalidated and I will compensate the company for the loss.

Signature of interviewer:

S-screening questionnaire

S1. [Circled by the interviewer] City of Respondent?

**First-tier city**	Beijing	1	Shanghai	2	Guangzhou	3
**Second-tier city**	Xi'an	4	Wuhan	5	Fuzhou	6
	Nanjing	7	Hangzhou	8	Chengdu	9
	Shenyang	10	Ji'nan	11	Zhengzhou	12
	Changsha	13				

S2. [Single answer] What level of hospital are you in?

Tertiary hospital	1	Continue
Others	2	Thank and end

S3. [Single answer] Which department are you in?

Rheumatology	1	Continue
Others	2	Thank and end

S4. [Single answer] What is your professional title?

Chief physician	1	Continue
Associate chief physician	2	Continue
Attending physician (5 years and above)	3	Continue
Others	4	Thank and end

S5. [Complete] How many **rheumatoid arthritis** patients do you receive on average **every week**?

**Rheumatoid arthritis** patients	___________(number)	≥30, continue	< 30, thank and end

S6. [Single answer] Do you personally formulate a therapeutic regimen for patients with rheumatoid arthritis?

Yes	1	Continue
No	2	Thank and end

S7. [Transverse single answer] How well do you know about the following drugs for rheumatoid arthritis?

Drug type		Knowledge about the drug
csDMARD	Methotrexate	□ Never heard □ Heard, but know nothing else<br>□ Know but not yet prescribed □ Occasional prescription<br>□ Conventional prescription
	Leflunomide	□ Never heard □ Heard, but know nothing else<br>□ Know but not yet prescribed □ Occasional prescription<br>□ Conventional prescription
	Sulfasalazine	□ Never heard □ Heard, but know nothing else<br>□ Know but not yet prescribed □ Occasional prescription<br>□ Conventional prescription
	Hydroxychloroquine	□ Never heard □ Heard, but know nothing else<br>□ Know but not yet prescribed □ Occasional prescription<br>□ Conventional prescription
bDMARD	Etanercept	□ Never heard □ Heard, but know nothing else<br>□ Know but not yet prescribed □ Occasional prescription<br>□ Conventional prescription
	Rituximab	□ Never heard □ Heard, but know nothing else<br>□ Know but not yet prescribed □ Occasional prescription<br>□ Conventional prescription
	Infliximab	□ Never heard □ Heard, but know nothing else<br>□ Know but not yet prescribed □ Occasional prescription<br>□ Conventional prescription
	Adalimumab	□ Never heard □ Heard, but know nothing else<br>□ Know but not yet prescribed □ Occasional prescription<br>□ Conventional prescription
	Tocilizumab	□ Never heard □ Heard, but know nothing else<br>□ Know but not yet prescribed □ Occasional prescription<br>□ Conventional prescription
tsDMARD	Tofacitinib	□ Never heard □ Heard, but know nothing else<br>□ Know but not yet prescribed □ Occasional prescription<br>□ Conventional prescription

Main Questionnaire

Part I: Number of Patients

Q1. [Complete] How many half-days do you receive patients every week? What is your weekly outpatient volume? How many rheumatoid arthritis patients do you receive every week?

No.	Item	Number
1	Number of days on duty per week	__________half-days
2	Outpatient volumn per week	______________patients
3	Number of **rheumatoid arthritis** patients visiting the outpatient per week	______________patients
		Q1.3≤Q1.2

Q2. [Complete] What is the proportion of newly diagnosed patients in the rheumatoid arthritis patients visiting the outpatient? What is the proportion of follow-up patients?

No.	Patient type	Total proportion=100%
1	Proportion of newly diagnosed patients	_____________%
2	Proportion of follow-up patients	_____________%

Q3. [Complete] How many hospitalized patients do you manage on average per month? How many rheumatoid arthritis patients are there?

No.	Item	Number
1	Number of inpatients per month	_______patients
2	Number of **rheumatoid arthritis inpatients** per month	_______patients
		Q3.2≤Q3.1

Part II: Diagnosis, Treatment and Follow-up of Rheumatoid Arthritis Patients at Outpatient

Q4. [Complete] How long does it take on average from the first visit to diagnosis for a rheumatoid arthritis patient?

Item	Time
Average time from the first visit to the diagnosis of rheumatoid arthritis	_____________month(s)

Q5. [Complete] What is the proportion of **newly diagnosed** rheumatoid arthritis patients who receive drug therapy at outpatient?

Item	Proportion≤100%
Proportion of patients receiving drug therapy	_____________%

Q6. [Complete] For **newly diagnosed rheumatoid arthritis** patients, what are the prescription proportions of the following first-line therapeutic regimens?

No.	Prescription drug regimen	Proportion
1	csDMARD alone	_____________%
2	One csDMARD + glucocorticoid	_____________%
3	One csDMARD + NSAIDs	_____________%
4	Combination of two csDMARDs	_____________%
5	bDMARD/tsDMARD alone	_____________%
6	Others, please specify_________	_____________%
		Total longitudinal proportion=100%

Q7. [Longitudinal multiple answers] Under what conditions you will not prescribe the following drugs?

Q8. [Complete] **[Selected 1/2/8 for any drug in Q7]** What is the examination indicator corresponding to the contraindication?

No.	Condition	csDMARD	TNFi	IL-6i	JAKi	Indicator
1	Severe renal insufficiency	□	□	□	□	□ Serum creatinine >__μmol/L<br>□ GFR<____ml/min
2	Severe hepatic insufficiency	□	□	□	□	ALT/AST>____u/L<br>GGT>____u/L
3	Pregnant/lactating women	□	□	□	□	
4	Alcoholism or alcoholic liver disease	□	□	□	□	
5	Peptic ulcer or ulcerative colitis	□	□	□	□	
6	Bone marrow suppression or existing blood dyscrasias	□	□	□	□	
7	Overt or laboratory evidence of immunodeficiency	□	□	□	□	WBC count<____*10^9/L
8	Allergic to csDMARD and its metabolites	□	□	□	□	
9	Interstitial lung disease	□	□	□	□	
10	Latent tuberculosis	□	□	□	□	
11	History of hepatitis B or C	□	□	□	□	
12	Others, please specify_________	□	□	□	□	

Q9. [Complete] What are the proportions of the following drugs in newly diagnosed rheumatoid arthritis patients who are prescribed with **csDMARD** as the first-line therapy? Oral administration or injection? What is the initial dose? What is the ideal dose? What is the proportion of patients who reach the ideal dose? What is the actual dose for most patients?

csDMARD	Proportion of patients	Route of administration	Initial dose	Ideal dose (minimum dose to achieve ideal curative effect)	Proportion of patients reaching ideal dose	Actual dose for most patients
1 Methotrexate	____%	□ Oral<br>□ Injection	Weekly dose:<br>□5mg<br>□7.5mg<br>□10mg<br>□12.5mg<br>□15mg<br>□___mg	Weekly dose:<br>□10mg<br>□12.5mg □15mg<br>□17.5mg □20mg<br>□22.5mg □25mg<br>□___mg	____%	Weekly dose:<br>□10mg<br>□12.5mg<br>□15mg<br>□17.5mg<br>□20mg<br>□22.5mg<br>□25mg<br>□___mg
2 Leflunomide	____%	□ Oral<br>□ Injection	Daily dose:<br>□5mg<br>□10mg<br>□15mg<br>□20mg<br>□___mg	Daily dose:<br>□10mg<br>□15mg □20mg<br>□25mg □___mg	____%	Daily dose:<br>□10mg<br>□15mg □20mg<br>□25mg<br>□___mg
3 Sulfasalazine	____%	□ Oral<br>□ Injection	Daily dose:<br>□1g □1.5g<br>□2g □2.5g<br>□3g □___g	Daily dose:<br>□1g □1.5g<br>□2g □2.5g<br>□3g □___g	____%	Daily dose:<br>□1g □1.5g<br>□2g □2.5g<br>□3g □___g
4 Hydroxychloroquine	____%	□ Oral<br>□ Injection	Daily dose:<br>□200mg<br>□300mg<br>□400mg<br>□___mg	Daily dose:<br>□200mg<br>□300mg □400mg<br>□___mg	____%	Daily dose:<br>□200mg<br>□300mg<br>□400mg<br>□___mg
5 Others______	____%	□ Oral<br>□ Injection	Daily dose:<br>_______mg	Daily dose:<br>_______mg	____%	Daily dose:<br>_______mg

Q10. [Single answer] For patients treated with **methotrexate**, under what conditions will folic acid be supplemented?

No.	Conditions of supplementation	Option
1	Folic acid is routinely supplemented as long as methotrexate is used.	□
2	Folic acid is supplemented when side effect occurs.	□
3	Folic acid is supplemented when **serious** side effect occurs.	□
4	Others, please specify__________	□

Q11. [Complete] What are the proportions of the following drugs in newly diagnosed rheumatoid arthritis patients who are prescribed with **bDMARD/tsDMARD**?

No.	bDMARD/tsDMARD	
1	Etanercept (Enbrel)	________%
2	Etanercept (Etanar)	________%
3	Rituximab (MabThera)	________%
4	Infliximab (Remicade)	________%
5	Adalimumab (Humira)	________%
6	Tocilizumab (Actemera)	________%
7	Tofacitinib (Xeljanz)	________%
8	Others, please specify__________	________%
		Total longitudinal proportion=100%

Q12. [Complete] How long will the first follow-up begin after starting first-line drug therapy for the newly diagnosed rheumatoid arthritis patients to evaluate the efficacy and adverse reactions?

Item	Time
Time of first follow-up	After______________weeks

Q13. [Multiple answers] Which of the following indicators do you mainly use to evaluate the efficacy of rheumatoid arthritis drugs?

Examination/evaluation method		Tick
1 Evaluation scale	1 DAS-28 score	□
	2 CDAI score	□
	3 SDAI score	□
	4 VAS score of patients	□
	5 Others, please specify__________	□
2 Laboratory examination	1 RF	□
	2 CRP	□
	3 ESR	□
	4 ACPA	□
	5 CCP	□
	6 Others, please specify__________	□
3 Imaging	1 X-ray	□
	2 Ultrasound	□
	3 CT	□
	4 MRI	□
	5 Others, please specify__________	□
4 Others, please specify__________		□

Q14. [Complete] After first-line treatment, what is the proportion of outpatients who achieve clinical remission? What are the proportions of low disease activity and moderate/high disease activity respectively?

No.	Patient type	Proportion
1	After first-line treatment: Patients with clinical remission	________%
2	After first-line treatment: Patients with low disease activity	________%
3	After first-line treatment: Patients with moderate/high disease activity	________%

Q15. [Complete] How do you adjust the subsequent therapeutic regimen for **rheumatoid arthritis patients who use different csDMARDs for first-line treatment without clinical remission**?

First-line	Subsequent therapeutic regimen	Proportion
One csDMARD	1 Maintain the original therapy	________%
	2 Increase dose	________%
	3 Add one csDMARD	________%
	4 Add two csDMARDs	________%
	5 Change to one bDMARD/tsDMARD	________%
	6 Add one bDMARD/tsDMARD	________%
	7 Others, please specify_________	________%
		Total longitudinal proportion=100%
Two csDMARDs	1 Maintain the original therapy	________%
	2 Increase dose	________%
	3 Change to another csDMARD	________%
	4 Add one csDMARD	________%
	5 Change to one bDMARD/tsDMARD	________%
	6 Add one bDMARD/tsDMARD	________%
	7 Others, please specify_________	________%
		Total longitudinal proportion=100%

Q16. How long will the first follow-up begin after adjustment of therapeutic regimen to evaluate the efficacy and adverse reactions? How often is the routine follow-up thereafter?

No.	Item	Time
1	The first follow-up time after adjustment of regimen	After __________weeks
2	Routine follow-up frequency	Every __________ weeks

Part IV: Drug Treatment for Inpatients with Rheumatoid Arthritis

Q17. [Multiple answers] What type of rheumatoid arthritis patients do you require hospitalization?

No.	Patient type	Tick
1	The patient's condition is still beyond control after drug treatment, thus hospitalization is required for standard treatment.	□
2	Consider surgical treatment	□
3	Combined with other complications	□
4	Others, please specify_________	□

Q18. [Complete] For **inpatients with rheumatoid arthritis**, what are the prescription proportions of the following drug therapeutic regimens?

No.	Prescription drug regimen	Total proportion=100%
1	csDMARD alone	____________%
2	One csDMARD + glucocorticoid	____________%
3	One csDMARD + NSAIDs	____________%
4	Combination of two csDMARDs	____________%
5	Combination of three csDMARDs	____________%
6	bDMARD/tsDMARD alone	____________%
7	csDMARD+bDMARD/tsDMARD	____________%
8	Others, please specify_________	____________%

Q19. [Complete] What are the proportions of the following drugs in rheumatoid arthritis inpatients who are prescribed with csDMARD?

Q20. [Transverse single answer] What are the recommended doses of different drugs?

No.	csDMARD	Total proportion≥100%	Dose
1	Methotrexate	_______%	Weekly dose: □<5mg □5-10mg □10-20mg □>20mg
2	Leflunomide	_______%	Daily dose: □<10mg □10-15mg □15-20m □>20mg
3	Sulfasalazine	_______%	Daily dose: □<1.5g □1.5-2g □2-3g □>3g
4	Hydroxychloroquine	_______%	Daily dose: □ <200mg □200-300mg □300-400mg □>400mg
5	Others, please specify__	_______%	

Q21. [Complete] What are the proportions of the following drugs in rheumatoid arthritis inpatients who are prescribed with bDMARD/tsDMARD?

No.	b/tsDMARD	Total proportion=100%
1	Etanercept (Enbrel)	_______%
2	Etanercept (Etanar)	_______%
3	Rituximab (MabThera)	_______%
4	Infliximab (Remicade)	_______%
5	Adalimumab (Humira)	_______%
6	Tocilizumab (Actemera)	_______%
7	Tofacitinib (Xeljanz)	_______%
8	Others, please specify_________	_______%

Part V: Adverse Reactions of csDMARD drugs

Q22. [Complete] What is the proportion of patients with side effects who use the following csDMARD drugs?

No.	Drug used	Proportion of patients with side effects
1	Methotrexate	__________%
2	Leflunomide	__________%
3	Sulfasalazine	__________%
4	Hydroxychloroquine	__________%

Q23. [Longitudinal multiple answers] What are the common side effects in patients taking the following csDMARD drugs?

Side effects			Methotrexate	Leflunomide	Sulfasalazine	Hydroxychloroquine
Patient's chief complaints	Gastrointestinal reaction	Abdominal pain	□	□	□	□
		Diarrhoea	□	□	□	□
		Constipation	□	□	□	□
		Nausea, vomiting	□	□	□	□
		Dental ulcer	□	□	□	□
		Acid regurgitation, abdominal distension and insufficient gastric motility	□	□	□	□
		Others______________________	□	□	□	□
	Behavioral expression	Insomnia	□	□	□	□
		Disgusted with the name/shape of the drug	□	□	□	□
		Memory loss, difficulty in concentration	□	□	□	□
		Anxiety, depression	□	□	□	□
		Others______________________	□	□	□	□
	Nonspecific manifestation	Weakness, fatigue	□	□	□	□
		Hair loss, rash	□	□	□	□
		Dryness-heat and chest burning	□	□	□	□
		Others______________________	□	□	□	□
Laboratory/imaging examination		Interstitial lung disease	□	□	□	□
		leukopenia	□	□	□	□
		Neutropenia	□	□	□	□
		Thrombocytopenia	□	□	□	□
		Impairment of liver and kidney functions (such as hematuresis)	□	□	□	□
		Others______________________	□	□	□	□

Q24. [Transverse single answer] Under what circumstances do you think the following side effects are serious? ***(Mild: the patient passively confirms and the side effect is tolerable; medium: the patient actively confirms, asks whether the treatment can be adjusted, and expresses tolerance after communication; serious: the patient actively confirms and complains of intolerance and insists on intervention or adjustment of treatment)***

Side effects			Severity of side effect
Patient's chief complaints	Gastrointestinal reaction	Abdominal pain	□ upon occurrence □ mild □ moderate □ serious □ in no case
		Diarrhoea	□ upon occurrence □ mild □ moderate □ serious □ in no case
		Constipation	□ upon occurrence □ mild □ moderate □ serious □ in no case
		Nausea, vomiting	□ upon occurrence □ mild □ moderate □ serious □ in no case
		Dental ulcer	□ upon occurrence □ mild □ moderate □ serious □ in no case
		Acid regurgitation, abdominal distension and insufficient gastric motility	□ upon occurrence □ mild □ moderate □ serious □ in no case
		Others________________________	□ upon occurrence □ mild □ moderate □ serious □ in no case
	Behavioral expression	Insomnia	□ upon occurrence □ mild □ moderate □ serious □ in no case
		Disgusted with the name/shape of the drug	□ upon occurrence □ mild □ moderate □ serious □ in no case
		Memory loss, difficulty in concentration	□ upon occurrence □ mild □ moderate □ serious □ in no case
		Anxiety, depression	□ upon occurrence □ mild □ moderate □ serious □ in no case
		Others________________________	□ upon occurrence □ mild □ moderate □ serious □ in no case
	Nonspecific manifestation	Weakness, fatigue	□ upon occurrence □ mild □ moderate □ serious □ in no case
		Hair loss, rash	□ upon occurrence □ mild □ moderate □ serious □ in no case
		Dryness-heat and chest burning	□ upon occurrence □ mild □ moderate □ serious □ in no case
		Others________________________	□ upon occurrence □ mild □ moderate □ serious □ in no case
Laboratory/ imaging examination	Interstitial lung disease		□ upon occurrence □ slight imaging change □ moderate imaging change □ serious imaging change □ in no case
	leukopenia		□ As long as it is lower than normal value □3*10^9/L □2*10^9/L □1*10^9/L □ in no case
	neutropenia		□ As long as it is lower than normal value □2*10^9/L □1*10^9/L □0.5*10^9/L □ in no case
	thrombocytopenia		□ As long as it is lower than normal value □100*10^9/L □50*10^9/L □25*10^9/L □ in no case
	Impairment of liver and kidney functions (such as hematuresis)		□ As long as it is higher than normal value □ 1-3 times normal value □ more than 3 times normal value □ in no case
	Others______________________		□ upon occurrence □ mild □ moderate □ serious □ in no case

Q25. [Single answer] Under which of the following circumstances do you think the patient is intolerant to csDMARD?

No.	Item	Tick
1	Any ≥1 serious side effect occurs	□
2	Any ≥2 serious side effects occur	□
3	Any ≥3 serious side effects occur	□
4	Others, please specify__________	□

Q26. [Complete] According to the above criteria for intolerance of csDMARD, what are the respective proportions of patients with intolerance when using the following drugs?

No.	Drug used	Proportion of patients with intolerance
1	Methotrexate	__________%
2	Leflunomide	__________%
3	Sulfasalazine	__________%
4	Hydroxychloroquine	__________%

Q27. [Horizontal Selection] Based on your clinical experience, what dose of the following three drugs is likely to make Chinese patients suffer from intolerance?

No.	Drug used	Dose
1	Methotrexate	Weekly dose: □>10mg □>15mg □>20mg
2	Leflunomide	Daily dose: □>10mg □>15mg □>20mg
3	Sulfasalazine	Daily dose: □>1.5g □>2g □>3g
4	Hydroxychloroquine	Daily dose: □>200mg □>300mg □>400mg

Q28. [Complete] What is the proportion you will adjust the regimen in patients with csDMARD intolerance?

Item	Proportion≤100%
Proportion of patients with therapeutic regimen adjusted	_____________%

Q29. [Complete] What is the proportion of patients who adopt various subsequent therapeutic regimens after intolerance occurs with the following csDMARD drugs?

csDMARD used	Subsequent therapeutic regimen		Proportion
1 Methotrexate	1 Discontinue the drug for observation		____________%
	2 Maintain the original dose and supplement folic acid regularly.		____________%
	3 Change to another csDMARD	1 Leflunomide	____________%
		2 Sulfasalazine	____________%
		3 Hydroxychloroquine	____________%
		4 Others____________	____________%
	4 Change to one bDMARD	1 Etanercept (Enbrel)	____________%
		2 Etanercept (Etanar)	____________%
		3 Rituximab (MabThera)	____________%
		4 Infliximab (Remicade)	____________%
		5 Adalimumab (Humira)	____________%
		6 Tocilizumab (Actemera)	____________%
	5 Change to one tsDMARD	1 Tofacitinib (Xeljanz)	____________%
	6 Others, please specify_________		____________%
			Total longitudinal proportion=100%
2 Leflunomide	1 Discontinue the drug for observation		____________%
	2 Change to another csDMARD	1 Methotrexate	____________%
		2 Sulfasalazine	____________%
		3 Hydroxychloroquine	____________%
		4 Others____________	____________%
	3 Change to one bDMARD	1 Etanercept (Enbrel)	____________%
		2 Etanercept (Etanar)	____________%
		3 Rituximab (MabThera)	____________%
		4 Infliximab (Remicade)	____________%
		5 Adalimumab (Humira)	____________%
		6 Tocilizumab (Actemera)	____________%
	4 Change to one tsDMARD	1 Tofacitinib (Xeljanz)	____________%
	5 Others, please specify_________		____________%
			Total longitudinal proportion=100%

Q30. [Longitudinal multiple answers] Which replacement regimen do you prefer when csDMARD intolerance occurs?

Regimen adjustment		**Gastrointestinal reaction** (abdominal pain/diarrhea/ constipation/ nausea, vomiting/ dental ulcer, etc.)	**Behavioral expression** (insomnia/ alopecia/ aversion to drug name and shape/ hypomnesis, etc.)	**Nonspecific manifestation** (weakness, fatigue/ alopecia, rash/ dryness-heat, chest burning, etc.)	**Laboratory examination** (white blood cells decreased/ myelosuppression/ interstitial lung disease/ liver and kidney function damage, etc.)
Maintain the original regimen (not intolerance)		□	□	□	□
Discontinue the drug for observation		□	□	□	□
Change to another csDMARD		□	□	□	□
Change to bDMARD	Etanercept (Enbrel)	□	□	□	□
	Etanercept (Etanar)	□	□	□	□
	Rituximab (MabThera)	□	□	□	□
	Infliximab (Remicade)	□	□	□	□
	Adalimumab (Humira)	□	□	□	□
	Tocilizumab (Actemera)	□	□	□	□
Change to tsDMARD	Tofacitinib (Xeljanz)	□	□	□	□
Others, please specify__________		□	□	□	□

Q31. [Complete] What is the proportion of your rheumatoid arthritis patients who voluntarily refuse methotrexate /csDMARD drug? If no one, please fill in "0"

Item	Proportion≤100%
Proportion of patients who voluntarily refuse methotrexate /csDMARD drug	_____________%

[Q31=0, skip to Q33]

Q32. [Multiple answers] What are the common reasons for patients to refuse methotrexate /csDMARD drug? ***(Select up to 3 items)***

No.	Reason	Select up to 3 items
1	csDMARD drug in use or ever used causes side effects.	□
2	The patient never used csDMARD drugs, but is worried about the side effect he/she heard of.	□
3	The patient never used csDMARD drugs, but is worried about the long-term side effect he/she heard of.	□
4	The patient has reproductive needs.	□
5	Tired of laboratory examination related to the drug used	□
6	Tired of taking medicine every week, or worried about forgetting to take medicine.	□
7	Questioning the curative effect	□
8	Others____	□

Q33. [Complete] What do you estimate is the proportion of patients who **stop or reduce their medication by themselves**? If no one, please fill in "0"

Item	Proportion≤100%
Proportion of patients who stop or reduce their medication	______________%

[Q33=0, skip to Q35]

Q34. [Multiple answers] What do you think are the most common reasons for patients to **stop or reduce their medication**? ***(Select up to 3 items)***

No.	Reason	Select up to 3 items
1	Joint symptoms have improved, but there are still mild side effects.	□
2	Symptoms are not controlled, but the side effects have more influence on the quality of life than the disease itself.	□
3	Worried about the possible long-term side effects	□
4	The patient has reproductive needs.	□
5	Tired of laboratory examination related to the drug used	□
6	Tired of taking medicine every week, or worried about forgetting to take medicine.	□
7	Questioning the curative effect	□
8	Others____	□

Part VI: Drug Evaluation

Q35. [Complete] How important are the following consideration factors when prescribing the bDMARD/tsDMARD drugs? Please rank by importance.

[Answer Q36-Q37 for bDMARD/tsDMARD drugs prescribed in Q11 and Q21]

Q36. [Complete] How do you think the following drugs perform against each consideration factor? Please score from 1 to 7, with 1 for “poor performance” and 7 for “very good performance”.

Q37. [Complete] What is your degree of satisfaction with the following drugs in treatment of rheumatoid arthritis? Please score from 1 to 7, with 1 for “very dissatisfied” and 7 for “very satisfied”.

No.	Consideration factor	Ranking of importance	Satisfaction score					
			Etanercept	Rituximab	Infliximab	Adalimumab	Tocilizumab	Tofacitinib
1	Quick action	Rank	□	□	□	□	□	□
2	Good effect in joint detumescence	Rank	□	□	□	□	□	□
3	Good effect in relieving pain	Rank	□	□	□	□	□	□
4	Good effect in alleviating anemia	Rank	□	□	□	□	□	□
5	Lasting curative effect	Rank	□	□	□	□	□	□
6	Good safety/few side effects	Rank	□	□	□	□	□	□
7	Sufficient medical evidence	Rank	□	□	□	□	□	□
8	Affordable medical cost	Rank	□	□	□	□	□	□
9	Others: ________	Rank	□	□	□	□	□	□
Overall satisfaction			□	□	□	□	□	□

Q38. [Single answer] In the cases of intolerance or poor curative effect, under what circumstances are the patients inclined to change to bDMARD or tsDMARD?

No.	Patient type	Option
1	After using one csDMARD, the patient is intolerant or the curative effect is poor.	□
2	After using two csDMARDs, the patient is intolerant or the curative effect is poor.	□
3	After using three csDMARDs, the patient is intolerant or the curative effect is poor.	□
4	Others, please specify__________	□

Q39. [Multiple answers] Which of the following types of rheumatoid arthritis patients do you think are suitable for **biologic monotherapy**?

Q40. [Transverse single answer] **[For the types of patients in Q39]** Which **bDMARD/tsDMARD** drug do you prefer for the following types of rheumatoid arthritis patients?

No.	Patient type	Monotherapy	bDMARD/tsDMARD					
			Etanercept	Rituximab	Infliximab	Adalimumab	Tocilizumab	Tofacitinib
1	csDMARD intolerant patients	□	□	□	□	□	□	□
2	Patients who do not comply with or refuse csDMARD therapy	□	□	□	□	□	□	□
3	Poor response to csDMARD	□	□	□	□	□	□	□
4	Rheumatoid arthritis complicated with other diseases	□	□	□	□	□	□	□
5	Others, please specify__________	□	□	□	□	□	□	□
6	None of the above	□						

Part VII: Patient Education

Q41. [Multiple answers] How do you think the education or knowledge popularization in rheumatoid arthritis patients will benefit clinical diagnosis and treatment?

No.	Benefits	Option
1	Improve the communication efficiency between doctor and patient	□
2	Improve patients' self-management	□
3	Enhance patients' cognition of the disease, thus improving their confidence in fighting against the disease	□
4	Others, please specify__________	□

Q42. [Single answer] Does your department regularly carry out patient education activities?

No.	Frequency	Option
1	Never	□
2	Occasionally (every six months or more)	□
3	Every quarter	□
4	Every 1-2 months	□
5	Others, please specify__________	□

[Q42=1, skip to Q45]

Q43. [Multiple answers] Which brands are currently carrying out educational activities for rheumatoid arthritis patients in your hospital?

No.	Company/brand	Option
1	Enbrel (Wyeth)	□
2	Etanar (CP Guojian)	□
3	MabThera (Roche)	□
4	Remicade (JOHNSON & JOHNSON)	□
5	Humira (Abbott)	□
6	Actemera (Roche)	□
7	Xeljanz (Pfizer)	□
8	Others, please specify__________	□

Q44. [Longitudinal multiple answers] **For the brands selected in Q43**, how are patient education activities conducted?

No.	Form of education	Enbrel (Wyeth)	Etanar (CP Guojian)	MabThera (Roche)	Remicade (JOHNSON & JOHNSON)	Humira (Abbott)	Actemra (Roche)	Xeljanz (Pfizer)
1	Patient Exchange	□	□	□	□	□	□	□
2	WeChat Official Account	□	□	□	□	□	□	□
3	Online expert consultation	□	□	□	□	□	□	□
4	Expert video online	□	□	□	□	□	□	□
5	Making patient education videos	□	□	□	□	□	□	□
6	Website-based science popularization	□	□	□	□	□	□	□
7	Paper publicity materials	□	□	□	□	□	□	□
8	Others, please specify__________	□	□	□	□	□	□	□

Q45. [Multiple answers] What are your most desired forms of effective patient education activities? ***(Select up to 3 items)***

No.	Patient education activities	Select up to 3 items
1	Patient Exchange	□
2	WeChat Official Account	□
3	Online expert consultation	□
4	Expert video online	□
5	Making patient education videos	□
6	Website-based science popularization	□
7	Paper publicity materials	□
8	Others, please specify__________	□

Q46. [Complete] What are the problems or your suggestions about patient education?

No.	Problems
1	____
